# Complete mitochondrial genome of *Spodoptera littoralis* (Lepidoptera: Noctuidae) from Egypt

**DOI:** 10.1080/23802359.2020.1870894

**Published:** 2021-02-09

**Authors:** Zaiyuan Li, Wenkai Wang, Lei Zhang

**Affiliations:** a Forewarning and Management of Agricultural and Forestry Pests, Hubei Engineering Technology Center, Institute of Entomological Science, College of Agriculture, Yangtze University, Jingzhou, China; b Agricultural Genomics Institute at Shenzhen, Chinese Academy of Agricultural Sciences, Shenzhen, China

**Keywords:** *Spodoptera littoralis*, mitochondrial genome, phylogenetic analysis, Noctuidae

## Abstract

The polyphagous cotton leafworm (*Spodoptera littoralis*) is one of the most destructive herbivorous insects worldwide. The present study reports the complete mitochondrial genome of *S. littoralis* collected from Egypt. The circular-mapping mitogenome was 15,408 bp in length with an overall A + T content of 81.1%, encoding a common set of 37 genes, including 13 protein-coding genes (PCGs), 22 tRNA genes, and two rRNA genes. Most PCGs were found to use conventional ATN as the start codon and TAN as the stop codon. The phylogenetic tree based on the nucleic acid sequences of 13 shared PCGs of 29 Noctuidae species revealed that *S. littoralis* and *Spodoptera litura* are sister species. The data in this study will be helpful to understand geographical genetic variations, phylogenetic relationships, and species identification of *S. littoralis*.

The cotton leafworm, *Spodoptera littoralis* (Boisduval, 1833) (Lepidoptera: Noctuidae), is considered one of the most destructive herbivorous pests because of its high reproductive rate and associated heavy losses to crops. *S. littoralis* can feed on more than 100 host plants, potentially resulting in yield losses of 50%, which is related to its larval foliage consumption activity (El-Sheikh et al. 2018; Garrido-Jurado et al. 2020). This species is widespread in Africa, Asia, and Europe and is also classified as a quarantine pest (EPPO; Pluschkell et al. 1998). Strong environmental adaptability makes it a threat to countries that currently have no record of it, and monitoring is important for *S. littoralis*. Unfortunately, *S. littoralis* have similar morphology to many *Spodoptera* species, especially the *Spodoptera* *litura*, which making their distinction by classical physical criteria difficult (Nagoshi et al. 2011). Therefore, it is a great challenge for the identification and correct differentiation of *S. littoralis*. Mitochondrial genes such as Cytochrome c oxidase subunit 1 (*cox1*) are often regarded as ideal markers because of their rich distribution, maternal inheritance, and high mutation rate, and thus they are widely used in species identification, genetic diversity, and molecular phylogeny (Dormann et al. 2008; Bernt et al. 2013; Bibi et al. 2020; Duan et al. 2020). Recently, the increasing number of whole mitochondrial genomes has greatly promoted phylogenetic studies in insects (Wu et al. 2016; Yang et al. 2019b; Zhou et al. 2020). In our study, to accurately monitoring and understand the phylogenetic position of *S. littoralis*, the complete mitogenome was determined.

In this study, the genomic DNA of *S. littoralis* was extracted from a male moth of an inbred strain established from eggs collected near Alexandria (31°07′52.6″N 29°56′'02.5″E) in Egypt in 2011. The voucher specimen’s genomic DNA was assigned with a unique code (AGIS-SL-EG-2011) and deposited at the Agricultural Genomics Institute, Chinese Academy of Agricultural Sciences, Shenzhen, China. Total genomic DNA was extracted using the Qiagen Genomic DNA kit (Cat. no.13323, Qiagen). DNA quality and concentration were determined using NanoDrop One UV-Vis spectrophotometer (Thermo Fisher Scientific). A total of 0.5 μg of genomic DNA was used to construct a 350-bp insert library, which was then sequenced using the Illumina NovaSeq 6000 platform with 150-bp paired-end reads. The mitogenome was assembled using the software NOVOPlasty v2.5.6 (Dierckxsens et al. 2017). Gene annotation was performed and circularity was checked using the web-based MITOS2 (http://mitos2.bioinf.uni-leipzig.de/index.py) (Bernt et al. 2013).

The complete mitogenome of *S. littoralis* (GenBank accession number MT816470) is 15,408 bp in length and contains 13 protein-coding genes (PCGs), 22 transfer RNA genes (tRNAs), two ribosomal RNA genes (rRNAs), and one non-coding A + T-rich region (putative control region). In the genome, the content of A + T is 81.1% (A 41.0%; T 40.0%), which represents a typical high A + T content among insect mitogenomes (Yang et al. 2019a). However, the A + T content varied among genes as follows: control region (96.0%), rRNAs (84.4 ∼ 85.0%), PCGs (72.4 ∼ 92.0%), and tRNAs (70.4 ∼ 91.2%). The negligible AT-skew (0.012) and negative GC-skew (−0.198) in the mitogenome of *S. littoralis* were found to be similar to those of other Lepidoptera insects (Cameron and Whiting 2008). The total length of the 13 PCGs was determined to be 11,150 bp. All PCGs use conventional ATN as the start codon, with one exception being CGA for *cox*1. These special structures of the start codon are a common feature of lepidopteran mitogenomes (Wu et al. 2016). Ten genes are terminated with a complete stop codon (TAA), one gene (*nad3*) has a complete stop codon (TAG), and two genes (*cox2* and *nad4*) end with an incomplete stop codon (T). Further, 22 tRNA genes ranged in size from 65 to 71 bp. All tRNAs harbor typical predicted secondary cloverleaf structures, except for *trnN* and *trnY*.

To validate the phylogenetic position of *S. littoralis*, we downloaded mitogenomes of 29 Noctuidae species and one outgroup species *Cydia pomonella* (Tortricidae) published in GenBank. Multiple nucleic acid sequence alignments of 13 PCGs were conducted using MAFFT v7.455 (Katoh and Standley 2013). The phylogenetic tree was constructed based on the maximum likelihood method using RAxML-NG v0.9.0 (Kozlov et al. 2019) with the best-fit model (GTR + F+R4) estimated by IQ-TREE v1.6.10 with parameter ‘-m MF’ (Nguyen et al. 2015). The bootstrap replicates were 1000 and the tree was visualized with FigTree v1.4.4 (http://tree.bio.ed.ac.uk/software/figtree/). The phylogenetic analysis showed that the *Spodoptera* genus was monophyletic, and *S. littoralis* and *S. litura* were sister species. In summary, the current mitogenome sequence will be useful for the phylogenetic analysis of this species (Figure 1). Furthermore, the mitochondrial DNA molecules within a cell are subject to evolutionary processes such as selection and drift. The high evolutionary rate of mitochondrial DNA makes it played an integral role in further revealing population structure and demographic history (Tikochinski et al. 2020; Johri et al. 2019; Hurst and Jiggins, 2005). *S. littoralis* is native to Africa and Israel (Yones et al. 2012), thus, the mitochondrial genome from Egypt provides the basis for further study of population genetics.

**Figure 1.**
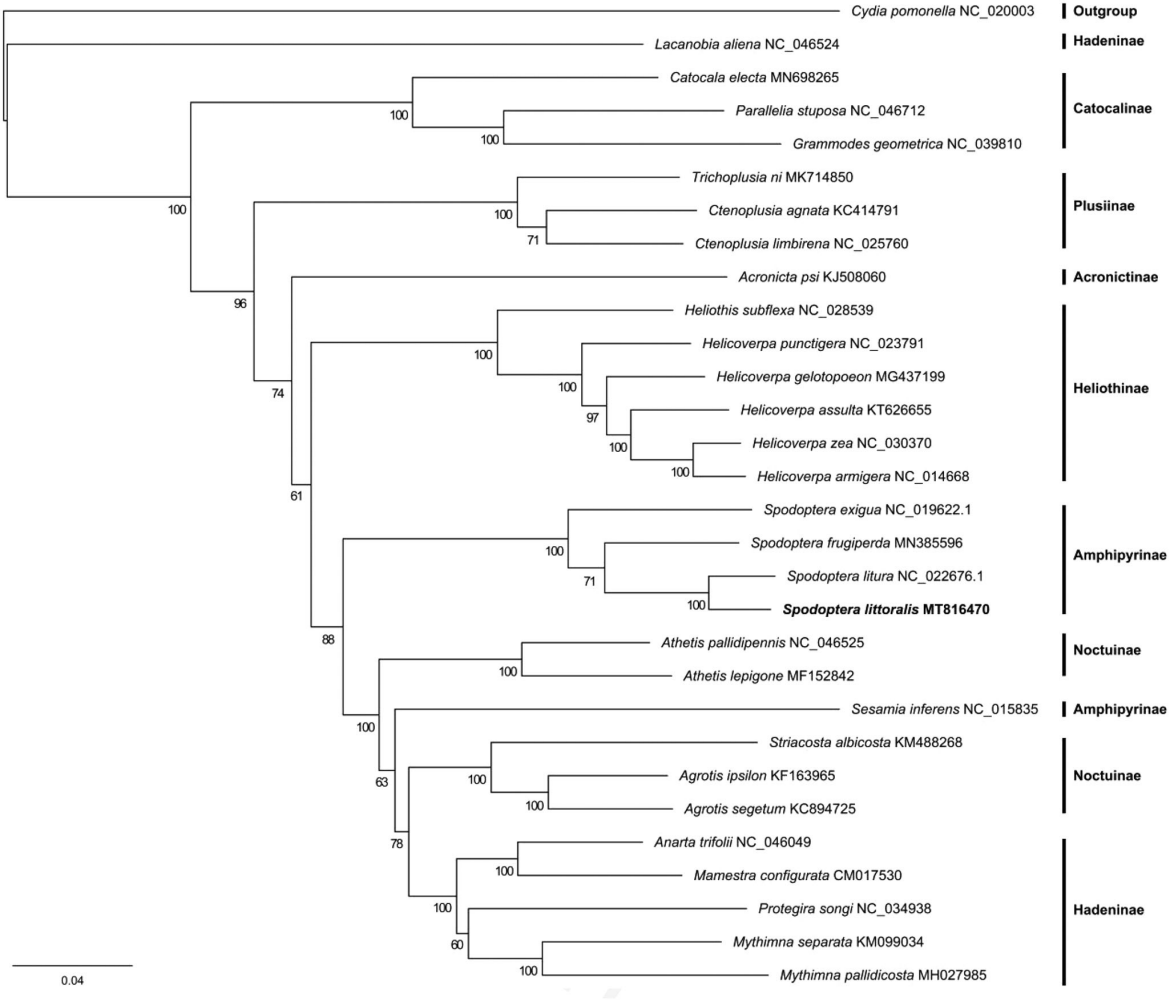
Phylogenetic tree of 30 species based on 13 concatenated mitochondrial protein-coding genes. *Cydia pomonella* was used as an outgroup.

## Data Availability

Mitogenome data supporting this study are openly available in GenBank at https://www.ncbi.nlm.nih.gov/nuccore/MT816470. Associated BioProject, BioSample, and SRA accession numbers are PRJNA678763, SAMN16812461, and SRR13083392, respectively.
